# PTL-PRS: an R package for transfer learning of polygenic risk scores with pseudovalidation

**DOI:** 10.1093/bioinformatics/btaf540

**Published:** 2025-09-24

**Authors:** Bokeum Cho, Seunggeun Lee

**Affiliations:** Graduate School of Data Science, Seoul National University, Seoul 08826, South Korea; Graduate School of Data Science, Seoul National University, Seoul 08826, South Korea

## Abstract

**Summary:**

Polygenic risk scores (PRSs) are essential tools for predicting individual phenotypic risk but often lack accuracy in non-European ancestry groups. Transfer Learning for Polygenic Risk Scores (TL-PRS) addresses this challenge by leveraging European PRSs to improve prediction in underrepresented ancestries but requires privacy-sensitive individual-level data and has low computational efficiency. Therefore, we introduce Pseudovalidated Transfer Learning for PRS (PTL-PRS), an extension of TL-PRS that incorporates pseudovalidation to eliminate the need for individual-level data and includes further software optimization. For pseudovalidation, PTL-PRS generates pseudo-summary statistics for training and validation and evaluates model performance with the pseudo-$R^{2}$ metric. To improve computational efficiency, PTL-PRS software was optimized with C++, blockwise early stopping, and direct genotype retrieval. Overall, PTL-PRS enhances usability while maintaining TL-PRS’s predictive performance.

**Availability and implementation:**

The *PTL.PRS* R package is publicly available on GitHub at https://github.com/bokeumcho/PTL.PRS. The summary statistics used in this paper are available in the public domain: UK Biobank (https://pheweb.org/UKB-TOPMed), PGS Catalog (https://www.pgscatalog.org), COVID-19 Host Genetics Initiative (https://www.covid19hg.org) and GenOMICC (https://genomicc.org/data).

## 1 Introduction

Polygenic risk scores (PRSs) estimate an individual’s genetic predisposition to traits or diseases on the basis of genome-wide association study (GWAS) data (Torkamani *et al.* 2018, Choi *et al.* 2020, Henches *et al.* 2023). They have also gained widespread application in biomedical research, including the assessment of shared etiology between phenotypes (Khera *et al.* 2018, Lambert *et al.* 2019). Despite their potential, PRSs are difficult to implement in clinical settings such as diagnostic tests and biomarker assessments because they do not perform as well in non-European ancestry, alongside challenges in clinical interpretability, calibrating to lifetime absolute risk and technical standards (Martin *et al.* 2019, Lewis and Vassos 2020, Weissbrod *et al.* 2022). To address these challenges, many cross-ancestry PRS methods have been developed to leverage large-scale datasets from European populations to enhance the prediction in underrepresented groups (Kachuri *et al.* 2024). Most of the methods use joint multi-ancestry Bayesian approach (Cai *et al.* 2021, Ruan *et al.* 2022, Zhou *et al.* 2023, Hoggart *et al.* 2024, Jin *et al.* 2024, Xu *et al.* 2025), which combines multi-ancestry summary statistics to estimate a shared effect distribution. In contrast, Transfer Learning for Polygenic Risk Scores (TL-PRS) (Zhao *et al.* 2022) treats cross-ancestry prediction as transfer learning, starting from any high-powered source PRS and applying deterministic gradient-descent updates to improve model generalizability and reduce the required sample size. However, the current implementation of TL-PRS relies on an individual-level tuning set to select the learning rate and early stopping iteration.

To improve the practical utility of TL-PRS, we introduce Pseudovalidated Transfer Learning for Polygenic Risk Scores (PTL-PRS), which integrates pseudovalidation into TL-PRS. While recent “auto” models like PRS-CSx-auto (Ruan *et al.* 2022), JointPRS-auto (Xu *et al.* 2025) bypass validation sets through implicit, in-model (empirical-Bayes) estimation, PTL-PRS enables explicit, summary statistics-based hyperparameter tuning and testing without a separate individual-level dataset. The approach builds upon the pseudosplitting framework established in PUMAS (Zhao *et al.* 2021) and extended in MegaPRS (Zhang *et al.* 2021), which enables partitioning a single set of summary statistics into pseudotraining and pseudovalidation sets. Model performance is evaluated using the pseudo-$R^{2}$ metric (Zhang *et al.* 2021), which facilitates the effective construction and assessment of TL-PRS models under limited data availability.

We implemented this strategy in an R package, PTL.PRS, a scalable software optimized for performance. PTL.PRS reimplements core training routines of TL-PRS in C++ to support multithreaded parallelism and incorporates techniques such as blockwise early stopping and index-based SNP retrieval from binary genotype files. These optimizations result in substantially reduced memory consumption and faster training times relative to those of the original TL-PRS framework.

## 2 Methods

**Figure 1.**
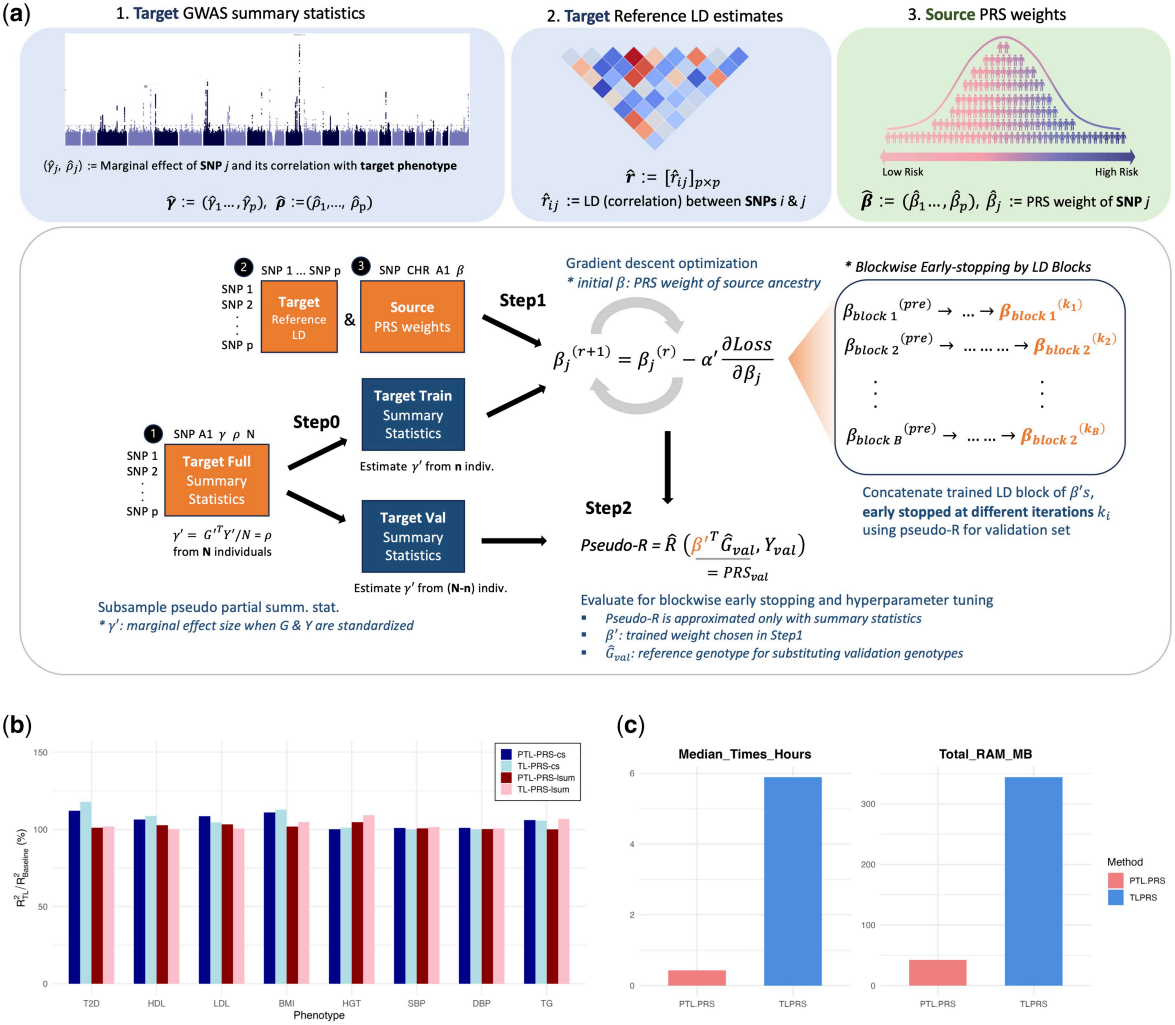
Overview of the PTL-PRS framework, its required inputs, and key results. (a) (*Top row) Three necessary inputs*: Target-ancestry GWAS summary statistics, Target population reference LD estimates and Source-ancestry PRS weights. *(Bottom row*) *Three main steps* of the PTL-PRS framework, using the inputs in the orange boxes (numbered as in top row) to fine-tune PRS weights of source population for the target population. *Step 0 (pseudosplitting)*: optional step to split the summary statistics data into training and validation sets. *Step 1 (training)*: gradient descent algorithm following TL-PRS procedures but with enhancements like blockwise early stopping (rightmost box) to optimize memory and time complexity. *Step 2 (evaluation)*: uses summary statistics to approximate the PRS-phenotype correlation (pseudo-$R^{2}$), guiding blockwise early stopping in Step 1 and hyperparameter tuning. (b) Relative accuracy of PTL-PRS and TL-PRS for different target phenotypes. For each phenotype, results are shown for PTL-PRS-cs and PTL-PRS-lsum together with the corresponding TL-PRS-cs and TL-PRS-lsum. Bar identities are indicated by their text labels in the legend at the top right. (c) Comparison of computational cost for training PTL-PRS and TL-PRS showing median execution time in hours and total RAM usage in MB. Benchmarking was conducted using three cores of an AMD EPYC 7542 (32-core) processor.

PTL-PRS consists of three main steps with three inputs, as shown in Fig. 1a. The required inputs are all summary statistics, including the following:

1. Target-ancestry GWAS summary statistics ($\hat{\gamma }$: marginal SNP effect sizes, $\hat{\rho }$: SNP–phenotype correlations),
2. Target population reference LD estimates ($\hat{r}$: pairwise SNP correlations), and
3. Source-ancestry PRS weights ($\hat{\beta }$).

Step 1 is based on TL-PRS (Zhao *et al.* 2022), while Steps 0 and 2 are adapted from the MegaPRS model (Zhang *et al.* 2021). The detailed workflow differences between TL-PRS and PTL-PRS are illustrated in Fig. 3, available as supplementary data at *Bioinformatics* online.

*Step 0 (pseudosplitting)* is an optional step applied when only one GWAS summary statistics dataset is available. It splits the summary statistics data into training and validation sets. With these pseudosplit summary statistics along with target LD estimates and source PRS weights, *Step 1 (training)* is performed, following TL-PRS procedures but with enhancements such as blockwise early stopping to optimize memory and time complexity. *Step 2 (evaluation)* involves the use of summary statistics to approximate the PRS-phenotype correlation (pseudo-$R^{2}$), guiding blockwise early stopping in Step 1 and hyperparameter tuning.

### 2.1 STEP 0: Subsampling pseudo-summary statistics

This step generates distinct summary statistics for training, validation, and testing (if necessary). It is achieved by emulating SNP marginal effect sizes (γ) through a random sampling technique (Zhao *et al.* 2021). The distribution used for this sampling, including variance estimation and detailed computational steps, is described in Equation (3), available as supplementary data at *Bioinformatics* online.

### 2.2 STEP 1: Transfer learning of polygenic risk scores

#### 2.2.1 *Recap of TL-PRS*

TL-PRS (Zhao *et al.* 2022) leverages transfer learning by initializing PRS weights from a source dataset and subsequently fine-tuning them via a gradient descent algorithm that is based on target ancestry data. The detailed equations are provided in Equation (1), available as supplementary data at *Bioinformatics* online.

#### 2.2.2 *Blockwise early stopping*

We improved the early stopping mechanism in TL-PRS by leveraging approximately independent linkage disequilibrium (LD) blocks (Berisa and Pickrell 2016). While TL-PRS originally used LD blocks to parallelize training, we extended this approach to include parallelized model validation, further enhancing computational efficiency. Each LD block is treated as an independent unit for training and validation. After each iteration of coefficient update, we compute pseudo-$R^{2}$ values for each block (see Step 2 and Equation (2), available as supplementary data at *Bioinformatics* online for details). If the pseudo-$R^{2}$ value for a given block decreases in subsequent iterations, coefficient updates for that block are terminated.

Unlike TL-PRS, which stores all intermediate coefficients across iterations and learning rates, PTL-PRS retains only the final set of coefficients per learning rate, significantly reducing storage usage. The final coefficients are concatenated across LD blocks, with each block potentially stopped at a different iteration because of blockwise early stopping. Additionally, pseudo$-R^{2}$ values are computed blockwise and aggregated to select the optimal learning rate, eliminating redundant computations for LD blocks that have already converged.

### 2.3 STEP 2: Calculation of pseudo-$R^{2}$

To eliminate reliance on individual-level data for model evaluation, we adopted a pseudo-$R^{2}$ implemented in MegaPRS (Zhang *et al.* 2021). This approach leverages standardized genotypic and phenotypic data to compute the Pearson correlation coefficient between observed and predicted phenotypes. The detailed formula for calculating pseudo-$R^{2}$ is provided in Equation (2), available as supplementary data at *Bioinformatics* online.

### 2.4 Computational optimization

To increase computational efficiency, we reimplemented core functions in C++, including binary file reading and coefficient updating. PTL-PRS directly accesses PLINK (.bed) genotype files through an index-based search implemented in C++ using Rcpp (Eddelbuettel and François 2011), eliminating the need for intermediate txt file conversions required by TL-PRS. Using RcppParallel (Allaire et al. 2025), we reimplemented the coefficient update function, which is the most time-consuming step, in C++ and parallelized it across LD blocks. To reduce memory usage, we divided the LD block input data, the main contributor to peak RAM consumption, into smaller chunks. These chunks are stored in queues, passed to the coefficient update function in parallel, and then removed from memory after processing.

## 3 Results

We evaluated the performance of PTL-PRS by (i) comparing it with the original TL-PRS across eight phenotypes, (ii) assessing the predictive accuracy for COVID-19 severity, and (iii) measuring enhancements in time and memory efficiency. We tested four models—PTL-PRS-cs, PTL-PRS-lsum, TL-PRS-cs, and TL-PRS-lsum—categorized in Table 1, available as supplementary data at *Bioinformatics* online. The prefixes “PTL-PRS” and “TL-PRS” indicate whether pseudovalidation was applied, whereas the suffixes “-cs” and “-lsum” specify the underlying PRS method: PRS-cs (Ge *et al.* 2019) or Lassosum (Mak *et al.* 2017).

### 3.1 Comparison of PTL-PRS and TL-PRS performance

We evaluated the effects of PTL-PRS on eight phenotypes in South Asian ancestry: Type 2 Diabetes (T2D), High Density Lipoprotein (HDL), Low Density Lipoprotein (LDL), Body Mass Index (BMI), Height (HGT), Systolic Blood Pressure (SBP), Diastolic Blood Pressure (DBP), and Triglycerides (TG). These results were compared with those obtained with the TL-PRS method. Target ancestry summary statistics were derived from 6588 South Asian UK Biobank participants, whereas source ancestry PRS weights were obtained from ExPRSweb (Ma *et al.* 2022) via the PGS Catalog (Lambert *et al.* 2021), trained on UK Biobank White British and MGI European samples. After reserving 10% of the data for testing, the remaining samples were pseudosplit into training and validation sets (9:1). To account for variability introduced by random pseudosplitting, PTL-PRS was run five times with different random seeds, and the results were averaged.

Relative accuracy, defined as the percentage increase in $R^{2}$ over the baseline European PRS model without fine-tuning, is illustrated in Fig. 1b. The PTL-PRS and TL-PRS models exhibited comparable performance levels for each trait. Detailed performance metrics are provided in Table 2, available as supplementary data at *Bioinformatics* online.

### 3.2 Improved predictive accuracy without individual-level data

We applied the PTL-PRS to construct polygenic risk scores (PRSs) for COVID-19, comparing hospitalized patients to the healthy population using COVID-19 Host Genetics Initiative release seven GWAS meta-analysis (The COVID-19 Host Genetics Initiative 2020). As individual-level validation or test data are unavailable, making TL-PRS inapplicable, we instead utilized publicly accessible summary statistics from East Asian (EAS; 2882 cases/31 200 controls), African (AFR; 2589 cases/123 225 controls) and South Asian ancestry groups (SAS; 1622 cases/47 612 controls) as the target data and those with European ancestry group (EUR; 32 519 cases/2 062 805 controls) to compute the source PRS. Two rounds of pseudosplitting produced training, validation, and test summary statistics in an 8:1:1 ratio. Given the limited case counts, we anticipated higher variability from pseudosplitting and therefore repeated the procedure 30 times with different random seeds for each target ancestry. We recommend using enough random seeds to control variability, aiming for a standard error of relative accuracy below 2 percentage points. Since Lassosum requires individual-level or separate training and validation summary statistics, we used only the PTL-PRS-cs model.

Owing to the lack of access to individual-level test data, performance was evaluated using pseudo-$R^{2}$, whose validity was assessed by comparing pseudo-$R^{2}$ and true-$R^{2}$ values across the eight UKBB traits (Table 3, available as supplementary data at *Bioinformatics* online). While pseudo-$R^{2}$ values were slightly inflated, consistent with findings reported in PUMAS (Zhao *et al.* 2021), the relative improvement patterns closely aligned with those of true-$R^{2}$, supporting pseudo-$R^{2}$ as a reliable metric for evaluating relative accuracy.

Figure 1, available as supplementary data at *Bioinformatics* online presents the relative accuracy of the COVID-19 PRS by target ancestry, defined as the percentage ratio of fine-tuned to baseline pseudo-$R^{2}$. On average, PTL-PRS-cs achieved a pseudo-$R^{2}$ increase of 4.38% (SD = 2.51) for EAS, 6.01% (SD = 4.97) for AFR, and 3.76% (SD = 11.11) for SAS. Higher ancestry-specific case counts improved model stability, reducing variability across seeds (EAS > AFR > SAS). Most seeds showed no improvement for SAS, likely due to the relatively high genetic similarity between South Asian and European ancestry groups compared with other ancestry groups (Lan *et al.* 2023). Detailed results are shown in Table 4, available as supplementary data at *Bioinformatics* online.

Moreover, we performed a severity-stratified analysis comparing critically ill patients with those exhibiting mild symptoms. Our analysis showed improved predictive performance for SAS using PTL-PRS but PTL-PRS analysis for EAS and AFR ancestry showed reduced improvement compared with the covid19hg-based results. It reflects a tradeoff between a sharper case-control contrast and smaller sample size (Fig. 2, available as supplementary data at *Bioinformatics*  online, Table 5, available as supplementary data at *Bioinformatics* online; see also Note 2, available as supplementary data at *Bioinformatics* online for details).

### 3.3 Enhanced computational efficiency

We developed the R package *PTL.PRS*, which optimizes the original TL-PRS framework via blockwise early stopping and computational optimization using C++. We benchmarked *PTL.PRS* against *TLPRS* (an R implementation of the original TL-PRS method) using 984 143 SNPs to predict LDL levels in 6500 individuals of South Asian ancestry. Benchmarks were created on 3 CPU cores over 30 repeated runs. *PTL.PRS* achieved a median execution time of 25.6 minutes, corresponding to a 13.7-fold speedup over *TLPRS*, as shown in Fig. 1c. Incorporating the additional pseudosplitting step for generating pseudo-summary statistics increased the median execution time by 8.01 minutes. In terms of resource efficiency, compared with *TLPRS*, *PTL.PRS* reduced total RAM usage by 8.15-fold.

## 4 Conclusion

We developed PTL-PRS, an enhanced version of the TL-PRS framework that eliminates the need for individual-level data during model validation. It broadens the framework’s applicability to settings where privacy concerns or data access limitations prevent the sharing of individual-level genotypes or phenotypes. Across eight common phenotypes, PTL-PRS demonstrated predictive accuracy comparable to that of TL-PRS while offering improved computational efficiency, positioning it as a robust and scalable alternative for data-limited contexts. When applied to COVID-19 data, the PTL-PRS yielded substantial gains in prediction accuracy only with summary statistics. These results highlight the potential of PTL-PRS as a practical solution for genetic risk prediction in scenarios with limited sample sizes and restricted data accessibility.

## Supplementary Material

btaf540_Supplementary_Data

## Data Availability

Linkage disequilibrium (LD) reference panel datasets for each target ancestry are available on Zenodo at https://doi.org/10.5281/zenodo.16786034. Genome-wide association summary statistics analyzed in this study are publicly available from UK Biobank (https://pheweb.org/UKB-TOPMed), the PGS Catalog (https://www.pgscatalog.org), the COVID-19 Host Genetics Initiative (https://www.covid19hg.org), and GenOMICC (https://genomicc.org/data), subject to the terms of each repository.
