# Supplementary material for: PTL-PRS: an R package for transfer learning of polygenic risk scores with pseudovalidation
Source: Bioinformatics. 2025 Sep 24;41(10):btaf540. doi: 10.1093/bioinformatics/btaf540 (PMC12529095; doi:10.1093/bioinformatics/btaf540)
Supplement: btaf540_Supplementary_Data [file btaf540_supplementary_data.docx]

**PTL-PRS: an R package for transfer learning of polygenic risk scores with pseudovalidation**

Supplementary Data

Bokeum Cho^1^, Seunggeun Lee^1,*^

^1^ Graduate School of Data Science, Seoul National University, Seoul, South Korea

**Table of Content**

- Supplementary Figure S1. Relative accuracy of PTL-PRS-cs for COVID-19 across 30 different random seeds by target ancestry, using pseudo-*R*
- Supplementary Figure S2. Relative accuracy of PTL-PRS-cs for COVID-19 severity across 30 different random seeds by target ancestry, using pseudo-*R*.
- Supplementary Figure S3. Workflow comparison of TL-PRS (top) and PTL-PRS (bottom)
- Supplementary Table S1. 4 Types of models used in analysis
- Supplementary Table S2. Baseline $R^{2}$, model $R^{2}$, and relative accuracy of PTL-PRS and TL-PRS for different methods and traits, averaged over five random pseudosplitting seeds
- Supplementary Table S3. Baseline $R^{2}$, model $R^{2}$, and relative accuracy of PTL-PRS for different methods and traits, comparing true and pseudo-$R^{2}$ metrics averaged over five random pseudosplitting seeds
- Supplementary Table S4. Baseline pseudo-$R^{2}$, model pseudo-$R^{2}$, and relative accuracy of PTL-PRS-cs for COVID-19 by target ancestry across 30 random pseudosplitting seeds
- Supplementary Table S5. Baseline pseudo-$R^{2}$, model pseudo-$R^{2}$, and relative accuracy of PTL-PRS-cs for COVID-19 severity by target ancestry across 30 random pseudosplitting seeds
- Supplementary Note 1. Model requirements of TL-PRS
- Supplementary Note 2. Analysis using the GenOMICC dataset
- Supplementary Equation 1. Recap of TL-PRS
- Supplementary Equation 2. Formula for pseudo-*R*
- Supplementary Equation 3. Formula for pseudosplitting

Supplementary Figure S1. Relative accuracy of PTL-PRS-cs for COVID-19 across 30 different random seeds by target ancestry, using pseudo-*R*. Each dot represents the relative accuracy for a different random seed.


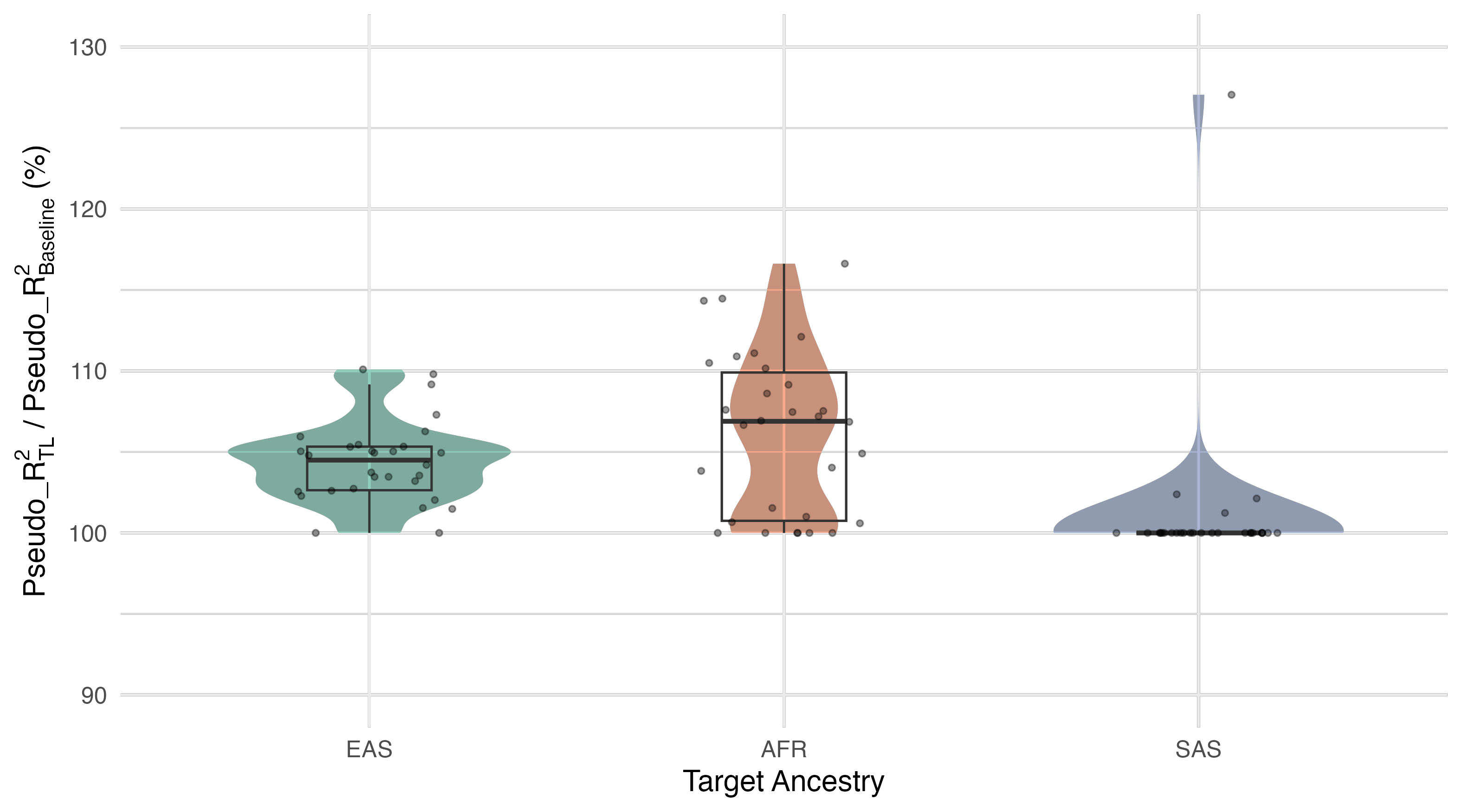


Supplementary Figure S2. Relative accuracy of PTL-PRS-cs for COVID-19 severity across 30 different random seeds by target ancestry, using pseudo-*R*. Each dot represents the relative accuracy for a different random seed


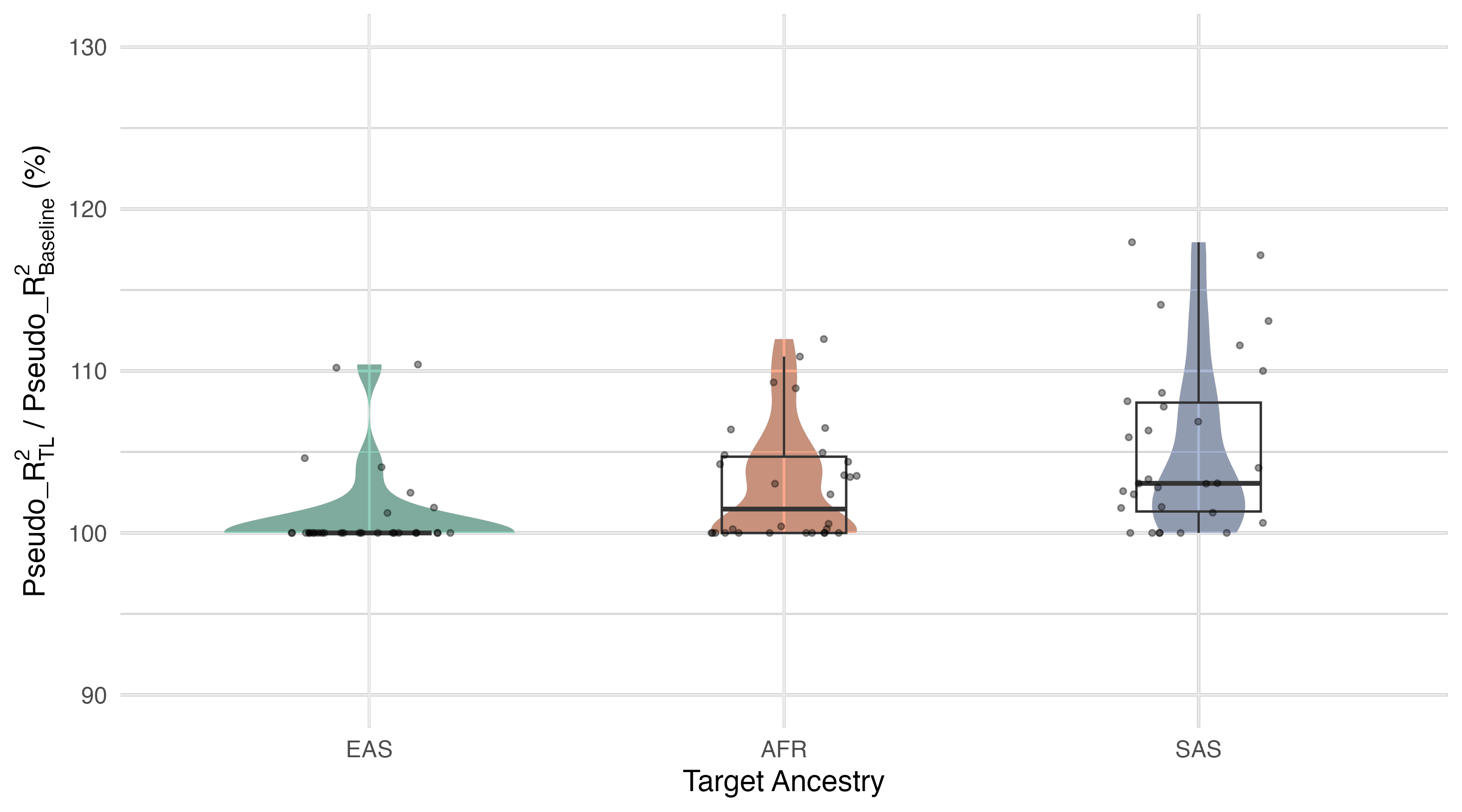


Supplementary Figure S3. Workflow comparison of TL-PRS (top) and PTL-PRS (bottom). PTL-PRS adds Step 0 (pseudosplitting) to replace individual-level validation data and approximates true-*R* with pseudo-*R*. It also employs blockwise early stopping, retaining only the best coefficient set, whereas TL-PRS stores all coefficient updates at every iteration.


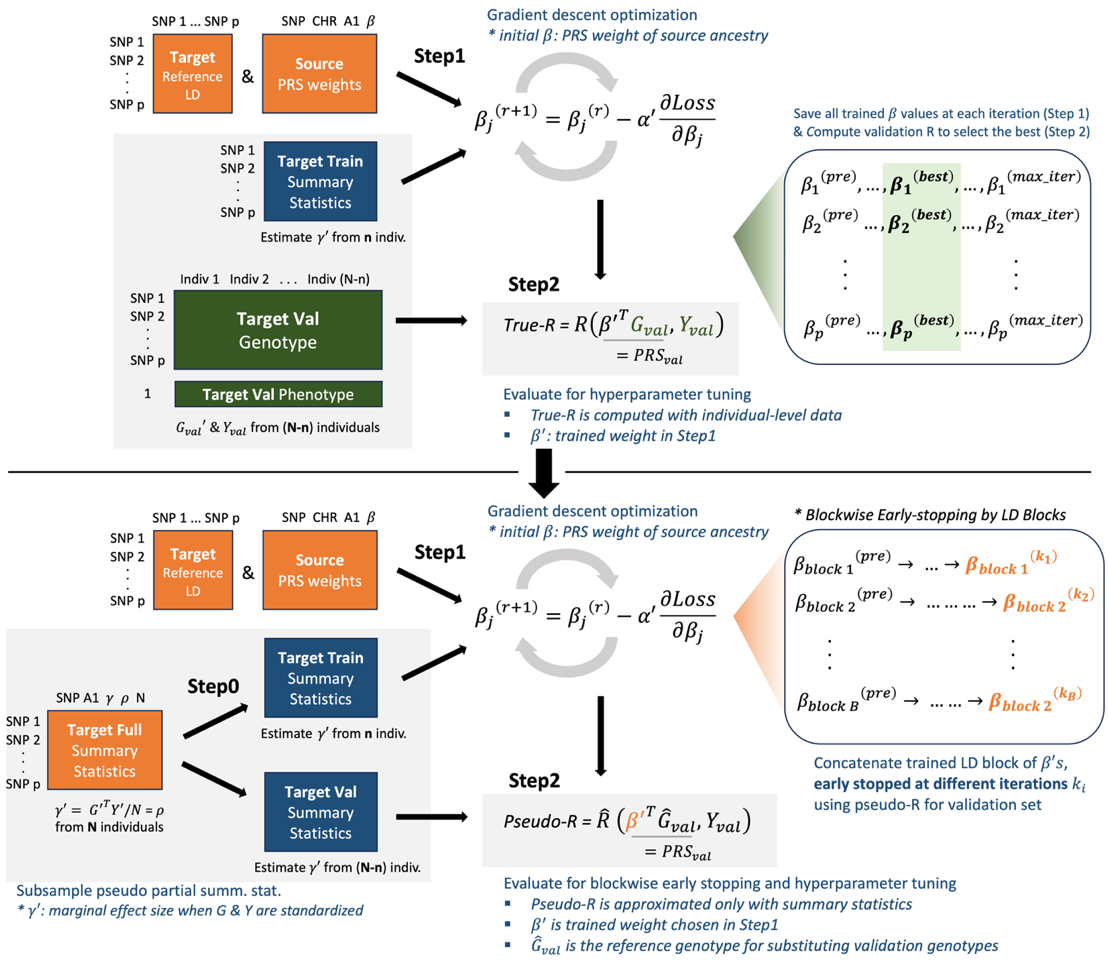


Supplementary Table S1. 4 Types of models used in analysis

|  |  | Pseudovalidation | |
| --- | --- | --- | --- |
|  |  | X | O |
| Baseline PRS | PRS-cs | TL-PRS-cs | PTL-PRS-cs |
|  | Lassosum | TL-PRS-Lsum | PTL-PRS-Lsum |

Supplementary Table S2. Baseline $\boldsymbol{R}^{\boldsymbol{2}}$, model $\boldsymbol{R}^{\boldsymbol{2}}$, and relative accuracy of PTL-PRS and TL-PRS for different methods and traits, averaged over five random pseudosplitting seeds

|  | PTL-PRS-cs | | | TL-PRS-cs | | |
| --- | --- | --- | --- | --- | --- | --- |
| Pheno | Baseline $R^{2}$ | Model $R^{2}$ | Rel. Acc.(%) | Baseline $R^{2}$ | Model $R^{2}$ | Rel. Acc.(%) |
| T2D | 0.00207 | 0.00231 | 112.03 | 0.00137 | 0.00161 | 117.94 |
| HDL | 0.03444 | 0.03664 | 106.40 | 0.02256 | 0.02454 | 108.78 |
| LDL | 0.00880 | 0.00955 | 108.50 | 0.01267 | 0.01326 | 104.67 |
| BMI | 0.01109 | 0.01231 | 111.02 | 0.00743 | 0.00839 | 113.01 |
| HGT | 0.01611 | 0.01612 | 100.10 | 0.01710 | 0.01730 | 101.17 |
| SBP | 0.02542 | 0.02565 | 100.89 | 0.02258 | 0.02258 | 100.00 |
| DBP | 0.01939 | 0.01958 | 101.01 | 0.01402 | 0.01402 | 100.00 |
| TG | 0.01665 | 0.01766 | 106.04 | 0.01134 | 0.01199 | 105.80 |
|  | PTL-PRS-lsum | | | TL-PRS-lsum | | |
| Pheno | Baseline $R^{2}$ | Model $R^{2}$ | Rel. Acc.(%) | Baseline $R^{2}$ | Model $R^{2}$ | Rel. Acc.(%) |
| T2D | 0.00389 | 0.00393 | 101.12 | 0.00356 | 0.00363 | 101.89 |
| HDL | 0.03246 | 0.03335 | 102.75 | 0.06819 | 0.06825 | 100.09 |
| LDL | 0.00903 | 0.00933 | 103.29 | 0.00889 | 0.00894 | 100.52 |
| BMI | 0.01765 | 0.01797 | 101.84 | 0.01345 | 0.01409 | 104.77 |
| HGT | 0.03034 | 0.03177 | 104.71 | 0.03926 | 0.04288 | 109.21 |
| SBP | 0.04945 | 0.04979 | 100.68 | 0.05012 | 0.05089 | 101.52 |
| DBP | 0.02964 | 0.02970 | 100.21 | 0.02147 | 0.02161 | 100.66 |
| TG | 0.02633 | 0.02633 | 100.01 | 0.03099 | 0.03310 | 106.82 |

Supplementary Table S3. Baseline $\boldsymbol{R}^{\boldsymbol{2}}$, model $\boldsymbol{R}^{\boldsymbol{2}}$, and relative accuracy of PTL-PRS for different methods and traits, comparing true and pseudo-$\boldsymbol{R}^{\boldsymbol{2}}$ metrics averaged over five random pseudosplitting seeds (true-$R^{2}$ values as in PTL-PRS results in Supplementary Table S2).

| Method | Pheno | Baseline $R^{2}$ | Model $R^{2}$ | Rel. Acc.(%) | Baseline pseudo-$R^{2}$ | Model  pseudo-$R^{2}$ | Rel. Acc.(%) |
| --- | --- | --- | --- | --- | --- | --- | --- |
| PTL-PRS-cs | T2D | 0.00207 | 0.00231 | 112.03 | 0.03063 | 0.03388 | 110.63 |
|  | HDL | 0.03444 | 0.03664 | 106.40 | 0.19616 | 0.20341 | 103.70 |
|  | LDL | 0.00880 | 0.00955 | 108.50 | 0.02186 | 0.02480 | 113.44 |
|  | BMI | 0.01109 | 0.01231 | 111.02 | 0.16829 | 0.17772 | 105.60 |
|  | HGT | 0.01611 | 0.01612 | 100.10 | 0.42652 | 0.44173 | 103.57 |
|  | SBP | 0.02542 | 0.02565 | 100.89 | 0.11937 | 0.13080 | 109.57 |
|  | DBP | 0.01939 | 0.01958 | 101.01 | 0.06008 | 0.06662 | 110.89 |
|  | TG | 0.01665 | 0.01766 | 106.04 | 0.10757 | 0.11586 | 107.71 |
| PTL-PRS-lsum | T2D | 0.00389 | 0.00393 | 101.12 | 0.02205 | 0.02325 | 105.42 |
|  | HDL | 0.03246 | 0.03335 | 102.75 | 0.21237 | 0.21663 | 102.01 |
|  | LDL | 0.00903 | 0.00933 | 103.29 | 0.04932 | 0.05204 | 105.51 |
|  | BMI | 0.01765 | 0.01797 | 101.84 | 0.14877 | 0.15055 | 101.19 |
|  | HGT | 0.03034 | 0.03177 | 104.71 | 0.26480 | 0.27929 | 105.47 |
|  | SBP | 0.04945 | 0.04979 | 100.68 | 0.09448 | 0.09829 | 104.04 |
|  | DBP | 0.02964 | 0.02970 | 100.21 | 0.05455 | 0.05663 | 103.81 |
|  | TG | 0.02633 | 0.02633 | 100.01 | 0.13130 | 0.13429 | 102.28 |

Supplementary Table S4. Baseline pseudo-$\boldsymbol{R}^{\boldsymbol{2}}$, model pseudo-$\boldsymbol{R}^{\boldsymbol{2}}$, and relative accuracy of PTL-PRS-cs for COVID-19 by target ancestry across 30 random pseudosplitting seeds

|  | EAS  (Baseline pseudo-$R^{2}$  = 0.005043) | | | AFR  (Baseline pseudo-$R^{2}$  = 0.00014851) | | | SAS  (Baseline pseudo-$R^{2}$  = 7.82.E-07) | |
| --- | --- | --- | --- | --- | --- | --- | --- | --- |
| Seed | Model pseudo-$R^{2}$ | Relative Accuracy (%) | Model pseudo-$R^{2}$ | | Relative Accuracy (%) | Model pseudo-$R^{2}$ | | Relative Accuracy (%) |
| 10 | 0.004986 | 100.00 | 0.0001647 | | 110.86 | 1.13.E-06 | | 145.04 |
| 20 | 0.005297 | 105.05 | 0.0001592 | | 107.16 | 2.94.E-07 | | 100.00 |
| 30 | 0.005284 | 104.79 | 0.0001508 | | 101.52 | 5.60.E-07 | | 100.00 |
| 40 | 0.005343 | 105.95 | 0.0001542 | | 103.81 | 6.14.E-07 | | 100.00 |
| 50 | 0.005145 | 102.03 | 0.0001597 | | 107.53 | 1.98.E-08 | | 100.00 |
| 60 | 0.005292 | 104.95 | 0.0001500 | | 101.00 | 5.54.E-08 | | 100.00 |
| 70 | 0.005359 | 106.27 | 0.0001641 | | 110.48 | 3.50.E-07 | | 100.00 |
| 80 | 0.005231 | 103.73 | 0.0001587 | | 106.89 | 7.99.E-07 | | 102.12 |
| 90 | 0.005174 | 102.60 | 0.0001558 | | 104.92 | 1.52.E-07 | | 100.00 |
| 100 | 0.005292 | 104.94 | 0.0001613 | | 108.58 | 1.01.E-07 | | 100.00 |
| 110 | 0.005158 | 102.28 | 0.0001461 | | 100.00 | 4.98.E-07 | | 100.00 |
| 120 | 0.005034 | 100.00 | 0.0001484 | | 100.00 | 9.94.E-07 | | 127.10 |
| 130 | 0.005171 | 102.55 | 0.0001588 | | 106.95 | 1.94.E-08 | | 100.00 |
| 140 | 0.005311 | 105.31 | 0.0001598 | | 107.57 | 3.16.E-08 | | 100.00 |
| 150 | 0.005312 | 105.33 | 0.0001596 | | 107.46 | 5.49.E-07 | | 100.00 |
| 160 | 0.005217 | 103.46 | 0.0001495 | | 100.67 | 1.06.E-06 | | 135.05 |
| 170 | 0.005254 | 104.20 | 0.0001494 | | 100.60 | 1.66.E-07 | | 100.00 |
| 180 | 0.005117 | 101.48 | 0.0001437 | | 100.00 | 1.70.E-07 | | 100.00 |
| 190 | 0.005552 | 110.09 | 0.0001471 | | 100.00 | 6.72.E-07 | | 100.00 |
| 200 | 0.005317 | 105.45 | 0.0001732 | | 116.61 | 2.98.E-07 | | 100.00 |
| 210 | 0.005204 | 103.20 | 0.0001698 | | 114.35 | 7.23.E-08 | | 100.00 |
| 220 | 0.005296 | 105.03 | 0.0001636 | | 111.07 | 1.75.E-08 | | 100.00 |
| 230 | 0.005221 | 103.55 | 0.0001650 | | 106.66 | 1.78.E-07 | | 100.00 |
| 240 | 0.005297 | 105.04 | 0.0001584 | | 100.00 | 7.92.E-07 | | 101.18 |
| 250 | 0.005181 | 102.73 | 0.0001477 | | 109.12 | 3.69.E-07 | | 100.00 |
| 260 | 0.005505 | 109.17 | 0.0001621 | | 114.45 | 6.49.E-07 | | 100.00 |
| 270 | 0.005537 | 109.80 | 0.0001700 | | 100.00 | 4.60.E-07 | | 100.00 |
| 280 | 0.005120 | 101.54 | 0.0001419 | | 112.11 | 8.01.E-07 | | 102.37 |
| 290 | 0.005411 | 107.29 | 0.0001665 | | 104.00 | 5.32.E-07 | | 100.00 |
| 300 | 0.005217 | 103.47 | 0.0001545 | | 105.79 | 5.34.E-07 | | 100.00 |
| **AVG** | **0.005261** | **104.38**  **(**$\boldsymbol{\pm}$**2.51)** | **0.000157** | | **106.01**  **(**$\boldsymbol{\pm}$**4.97)** | **4.31.E-07** | | **103.76**  **(**$\boldsymbol{\pm}$**11.11)** |

Supplementary Table S5. Baseline pseudo-$\boldsymbol{R}^{\boldsymbol{2}}$, model pseudo-$\boldsymbol{R}^{\boldsymbol{2}}$, and relative accuracy of PTL-PRS-cs for COVID-19 severity by target ancestry across 30 random pseudosplitting seeds

|  | EAS  (Baseline pseudo-$R^{2}$  = 0.00304445) | | | AFR  (Baseline pseudo-$R^{2}$  = 0.00099091) | | | SAS  (Baseline pseudo-$R^{2}$  = 0.00138870) | |
| --- | --- | --- | --- | --- | --- | --- | --- | --- |
| Seed | Model pseudo-$R^{2}$ | Relative Accuracy (%) | Model pseudo-$R^{2}$ | | Relative Accuracy (%) | Model pseudo-$R^{2}$ | | Relative Accuracy (%) |
| 10 | 0.002867 | 100.00 | 0.0010542 | | 106.38 | 0.0014245 | | 102.58 |
| 20 | 0.002920 | 100.00 | 0.0009159 | | 100.00 | 0.0014309 | | 103.04 |
| 30 | 0.002996 | 100.00 | 0.0010344 | | 104.39 | 0.0015016 | | 108.13 |
| 40 | 0.003361 | 110.40 | 0.0010551 | | 106.48 | 0.0014110 | | 101.61 |
| 50 | 0.002798 | 100.00 | 0.0009120 | | 100.00 | 0.0014969 | | 107.79 |
| 60 | 0.003092 | 101.57 | 0.0009585 | | 100.00 | 0.0013887 | | 100.00 |
| 70 | 0.002969 | 100.00 | 0.0009757 | | 100.00 | 0.0015843 | | 114.08 |
| 80 | 0.002757 | 100.00 | 0.0010258 | | 103.52 | 0.0015495 | | 111.58 |
| 90 | 0.003168 | 104.06 | 0.0010988 | | 110.88 | 0.0014765 | | 106.32 |
| 100 | 0.002875 | 100.00 | 0.0010330 | | 104.25 | 0.0015088 | | 108.65 |
| 110 | 0.002994 | 100.00 | 0.0010262 | | 103.56 | 0.0015704 | | 113.08 |
| 120 | 0.003120 | 102.48 | 0.0010400 | | 104.95 | 0.0014313 | | 103.07 |
| 130 | 0.003030 | 100.00 | 0.0009531 | | 100.00 | 0.0014347 | | 103.31 |
| 140 | 0.002718 | 100.00 | 0.0009492 | | 100.00 | 0.0014218 | | 102.39 |
| 150 | 0.003185 | 104.62 | 0.0009714 | | 100.00 | 0.0014101 | | 101.54 |
| 160 | 0.002965 | 100.00 | 0.0009965 | | 100.57 | 0.0014707 | | 105.91 |
| 170 | 0.002900 | 100.00 | 0.0010386 | | 104.81 | 0.0014311 | | 103.06 |
| 180 | 0.002960 | 100.00 | 0.0009932 | | 100.23 | 0.0013884 | | 100.00 |
| 190 | 0.003020 | 100.00 | 0.0009855 | | 100.00 | 0.0013973 | | 100.62 |
| 200 | 0.002763 | 100.00 | 0.0009871 | | 100.00 | 0.0015276 | | 110.00 |
| 210 | 0.002675 | 100.00 | 0.0009483 | | 100.00 | 0.0014061 | | 101.25 |
| 220 | 0.002978 | 100.00 | 0.0009949 | | 100.40 | 0.0013657 | | 100.00 |
| 230 | 0.002993 | 100.00 | 0.0010251 | | 103.45 | 0.0013775 | | 100.00 |
| 240 | 0.003000 | 100.00 | 0.0010794 | | 108.93 | 0.0014841 | | 106.87 |
| 250 | 0.003355 | 110.21 | 0.0010210 | | 103.04 | 0.0016268 | | 117.15 |
| 260 | 0.003082 | 101.25 | 0.0009934 | | 100.25 | 0.0013700 | | 100.00 |
| 270 | 0.002836 | 100.00 | 0.0009850 | | 100.00 | 0.0013885 | | 100.00 |
| 280 | 0.002851 | 100.00 | 0.0010830 | | 109.30 | 0.0014277 | | 102.81 |
| 290 | 0.002910 | 100.00 | 0.0011095 | | 111.97 | 0.0014445 | | 104.02 |
| 300 | 0.002773 | 100.00 | 0.0010145 | | 102.38 | 0.0016379 | | 117.94 |
| **AVG** | **0.002964** | **101.15**  **(**$\boldsymbol{\pm}$**2.76)** | **0.001009** | | **102.99**  **(**$\boldsymbol{\pm}$**3.62)** | **0.0014595** | | **105.23**  **(**$\boldsymbol{\pm}$**5.27)** |

**Supplementary Note 1. Model requirements of TL-PRS**

In the original TL-PRS paper [1], Supplementary Table S1 outlining model requirements for TL-PRS and TL-PRS(ind) states that TL-PRS does not require individual-level data for the validation step. However, the publicly available implementation (the TLPRS R package) does require individual-level data as input via the *test_file* argument. To resolve this discrepancy, we integrated a pseudovalidation technique into TL-PRS.

| Methods | Training dataset | Validation dataset | Testing dataset |
| --- | --- | --- | --- |
| TL-PRS | Only requires summary statistics | Individual-level data are recommended | Requires individual-level data to assess prediction performance |
| TL-PRS (ind) | Requires individual-level data | Requires individual-level data | Requires individual-level data to assess prediction performance |

Supplementary Table S1 from TL-PRS paper [1]: the model requirements of TL-PRS and TL-PRS (ind).

**Supplementary Note 2. Analysis using the GenOMICC dataset**

We applied PTL-PRS to construct polygenic risk scores (PRSs) for COVID-19 severity in East Asian, African and South Asian ancestry groups, using GenOMICC Release 2 GWAS summary statistics [2]. In this analysis, cases were critically ill patients and controls were individuals with mild symptoms. This contrasts with the COVID-19 Host Genetics Initiative (covid19hg) B2 cohorts, which defines cases as hospitalized patients and controls as population-based healthy individuals. The GenOMICC definition yields a smaller sample size but reduces case–control imbalance.

We used publicly available summary statistics from East Asian (EAS; 274 cases / 366 controls), African (AFR; 440 cases /1,350 controls) and South Asian (SAS; 788 cases / 3,793 controls) individuals as the target data, and from European individuals (EUR; 5,989 cases / 42,891 controls) as the source data for computing PRS weights. Following the same analysis protocol as for covid19hg, we performed two rounds of pseudosplitting to generate distinct training, validation, and test summary statistics in an 8:1:1 ratio. This process was repeated with 30 different random seeds to produce independent models.

Across these replicates, PTL-PRS-cs achieved an average increase of 1.15% in pseudo-$R^{2}$ (SD = 2.76) for EAS, 2.99% (SD = 3.62) for AFR and 5.23% (SD = 5.27) for SAS. The larger improvement in SAS ancestry suggests that mitigation of case–control imbalance outweighed losses from reduced sample size, whereas severely limited case count in EAS and AFR likely constrained improvements despite the sharper phenotypic contrast. Detailed results are provided in Supplementary Figure S2 and Supplementary Table S5.

**Supplementary Equation 1. Recap of TL-PRS**

A Polygenic Risk Score (PRS) is formulated as the aggregate of estimated effects across all genetic variants relevant to a specific phenotype. For a given individual denoted as *i*, the PRS can be calculated as following:

$$PRS_{i}=\sum_{j=1}^{M} \hat{\beta}_{j}G_{ij}$$

where M represents the total number of variants, $G_{ij}$ denotes the genotype of genetic variant *j*, and $\hat{\beta}_{j}$ is the marginal effect size of the variant *j*.

$$\begin{aligned} \boldsymbol{Y}=\sum_{j=1}^{M} G_{j}\beta_{j}+\varepsilon=\sum_{j=1}^{M} G_{j}(\beta_{j}^{pre}+\tau_{j})+\varepsilon\boldsymbol{\#}(1) \end{aligned}$$

$$\begin{aligned} Loss=\left( \boldsymbol{Y}-\sum_{j=1}^{M} G_{j}\beta_{j} \right)^{2}\#(2) \end{aligned}$$

$$\begin{aligned} \beta_{j}^{(r+1)}=\beta_{j}^{(r)}-\alpha^{'}\frac{\partial Loss}{\partial\beta_{j}}=\beta_{j}^{(r)}+2\alpha^{'}G_{j}^{T}(\boldsymbol{Y}-\boldsymbol{G}\beta^{(r)})\#(3) \end{aligned}$$

The equation 1 refers to the model form, equation 2 shows the loss function, and the equation 3 is a formula to estimate the next coefficient of variant *j*, given the current estimate $\beta_{j}^{(r)}$. During the model fitting phase, summary statistics of the target group can be utilized to estimate $\boldsymbol{G}^{T}\boldsymbol{Y}$ and$\boldsymbol{G}^{T}\boldsymbol{G}$ can be derived from the public reference datasets, such as the 1000 Genomes Project. The learning rate $\alpha=2\alpha^{'}$ can be chosen as parameter among the values the user offers, based on the validation dataset to optimize prediction accuracy. The default grid of learning rate is $min\left( \frac{\{1,10,100,1000\}}{n(SNPs)},1 \right)$.

**Supplementary Equation 2. Formula for pseudo-*R***

Assuming that both the genotype and phenotype data are standardized, the correlation between observed and predicted phenotypes for the individuals used in computing the validation or test summary statistics can be expressed as:

$$R=\frac{\beta^{'T}\boldsymbol{X}^{T}\boldsymbol{Y}}{\sqrt{n(\beta^{'T}\boldsymbol{X}^{T}\boldsymbol{X}\beta^{'})}} ,$$

where $\beta^{'}$ represents the vector of estimated effect sizes for a training model and $\boldsymbol{X}$ is the standardized form of genotype matrix $\boldsymbol{G}$.

Analogous to the model-fitting phase in TL-PRS, $\boldsymbol{X}^{T}\boldsymbol{Y}$ can be estimated with SNP-phenotype correlations ($\hat{\rho}$) from the validation summary statistics of the target phenotype, and $\boldsymbol{X}^{T}\boldsymbol{X}$ can be substituted with SNP-wise correlation ($\hat{r}$) from public reference dataset.

**Supplementary Equation 3. Formula for pseudosplitting**

The goal is to mimic marginal SNP effect sizes $\boldsymbol{\gamma}={(\gamma_{1},\gamma_{2},\ldots,\gamma_{m})}^{T}=\boldsymbol{G}^{T}\boldsymbol{Y}/n$, which equals to the correlation between SNP *j* and the phenotype, of distinct sample sets for train and test. Here, we assume $G$ and $Y$ are centered. $V$ refers to the variance of $\boldsymbol{G}^{T}\boldsymbol{Y}$. We obtain the following estimates from training (A) and test (B) sets:

- Estimate of $\boldsymbol{\gamma}$ from $n_{A}$ training samples:

$$\frac{\boldsymbol{G}_{\boldsymbol{A}}^{T}\boldsymbol{Y}_{\boldsymbol{A}}}{n_{A}}\sim\mathcal{N}\left( \frac{\boldsymbol{G}^{T}\boldsymbol{Y}}{n}, \frac{n_{B}}{n_{A}}\cdot\frac{\boldsymbol{V}}{n} \right)$$

- Estimate of $\boldsymbol{\gamma}$ from $n_{B}$ test samples:

$$\frac{\boldsymbol{G}_{\boldsymbol{B}}^{\boldsymbol{T}}\boldsymbol{Y}_{\boldsymbol{B}}}{n_{B}}=\frac{\boldsymbol{G}^{\boldsymbol{T}}\boldsymbol{Y-}\boldsymbol{G}_{\boldsymbol{A}}^{T}\boldsymbol{Y}_{\boldsymbol{A}}}{n_{B}}$$

To account for linkage disequilibrium, we follow Zhu and Stephens [3] and set:

$$\boldsymbol{V}=\boldsymbol{G}^{\boldsymbol{T}}\boldsymbol{G}$$

If $X^{'}$ denotes the standardized genotypes of a reference panel of size $n^{'}\times m$, we approximate $V$ by:

$$\boldsymbol{V} \approx\boldsymbol{X}^{\boldsymbol{'T}}\boldsymbol{X}^{\boldsymbol{'}}\frac{n}{n'}$$

Hence, we achieve the desired sampling by defining:

$$\frac{\boldsymbol{G}_{\boldsymbol{A}}^{T}\boldsymbol{Y}_{\boldsymbol{A}}}{n_{A}}=\frac{\boldsymbol{G}^{T}\boldsymbol{Y}}{n} +\sqrt{\frac{n_{B}}{n_{A}}}\frac{\boldsymbol{X}^{'T}}{\sqrt{n'}} g ,$$

where $g$ is a vector of length $n'$ with elements drawn from a standard Gaussian distribution.

**References**

1. Zhao, Z., et al., *The construction of cross-population polygenic risk scores using transfer learning.* The American Journal of Human Genetics, 2022. **109**(11): p. 1998-2008.

2. Kousathanas, A., et al., *Whole-genome sequencing reveals host factors underlying critical COVID-19.* Nature, 2022. **607**(7917): p. 97-103.

3. Zhu, X. and M. Stephens, *Bayesian large-scale multiple regression with summary statistics from genome-wide association studies.* The Annals of Applied Statistics, 2017. **11**(3).
